# A Rare and Challenging Ectopic Variceal Hemorrhage: A Case Report

**DOI:** 10.3390/reports8010018

**Published:** 2025-02-06

**Authors:** Christopher Pavel, Oana Mihaela Plotogea, Ecaterina Mihaela Rinja, Cosmin-Viorel Bogu, Andrei Turcescu

**Affiliations:** 1 Department 5, Carol Davila University of Medicine and Pharmacy, 050474 Bucharest, Romania; 2 Department of Gastroenterology, Clinical Emergency Hospital of Bucharest, 014461 Bucharest, Romania

**Keywords:** cirrhosis, ectopic varices, variceal bleeding, endoscopy, case report

## Abstract

**Background and Clinical Significance:** Ectopic variceal bleeding is a rare, but regrettably life-threatening, complication of hepatic cirrhosis. There is no standardized approach to this life-threatening event due to the absence of randomized controlled trials. Prompt identification of the bleeding site is crucial for timely hemostasis using endoscopic, radiologic or surgical methods. **Case presentation:** Throughout this paper, we present the case of a 52-year-old patient with decompensated alcoholic cirrhosis, who was admitted for melena. Upper and lower endoscopy failed to identify the source of bleeding. Ultimately, an evaluation with endoscopic capsule identified ileal varices. The patient was referred to surgery and the outcome was successful. We approached the diagnostic and therapeutic arsenals in managing ectopic varices. **Conclusions:** Although ectopic variceal bleeding has a substantial potential for fatal outcomes, prompt intervention in a multidisciplinary team could be the key for patient salvation.

## 1. Background and Clinical Significance

Variceal hemorrhage is regarded as a critical decompensating event in cirrhotic patients which severely impacts the vital prognosis, leading to a 5-year mortality rate of 80% when associated with other complications (ascites, hepatic encephalopathy) [1]. Management of esophagogastric variceal bleeding is clearly defined in the latest guidelines, but up to 5% of variceal hemorrhages occur from other sites; these are called ectopic varices [2]. Currently, there is no standardized approach to this potentially fatal event due to the heterogeneity of presentations and lack of randomized controlled trials. Immediate bleeding site identification is vital in order to proceed to hemostasis, either by endoscopic, radiologic or surgical techniques. Despite the significant advancements made in radiology and endoscopy, both in diagnostic and therapeutic capabilities, exploring ileo-jejunal varices and accurately assessing their true prevalence remain challenging.

We present the case of a patient with challenging ectopic variceal hemorrhage where the use of advanced radiologic and endoscopic technologies, alongside a multidisciplinary approach, was of utmost importance in order to ensure the best possible care.

## 2. Case Presentation

A 52-year-old female patient known with decompensated alcoholic cirrhosis (Child-Pugh C) and recurrent episodes of melena was transferred to our emergency department for another bleeding episode. She had previously been examined in another hospital unit through repeated upper and lower endoscopy procedures but without identification of any potential bleeding lesions.

The initial clinical examination revealed that the patient was in stable condition with a mild flapping tremor, but generally alert and oriented. Systolic blood pressure was 120 mmHg and tachycardia was noted (105 bpm, shock index 0.87). Lab tests showed moderate anemia with a hemoglobin level of 7.7 g/dL. We decided to repeat the endoscopic examinations and the EGD showed normal mucosa up to duodenum III. No varices had been detected even if other signs of portal hypertension (ascites, splenomegaly, thrombocytopenia) were present. During colonoscopy, multiple blood clots were observed, mainly in the ascending colon, cecum and terminal ileum. However, no potential bleeding site was detected. Therefore, it was determined that the site of bleeding was likely the small bowel, and further investigation was initiated. Unfortunately, after several hours from admission when the patient was apparently stable, hemodynamic instability (shock index > 1), rapid clinical deterioration and altered mental status occurred. The patient was consequently admitted into the ICU department with a high APACHE II score of 30 points.

**Figure 1:**
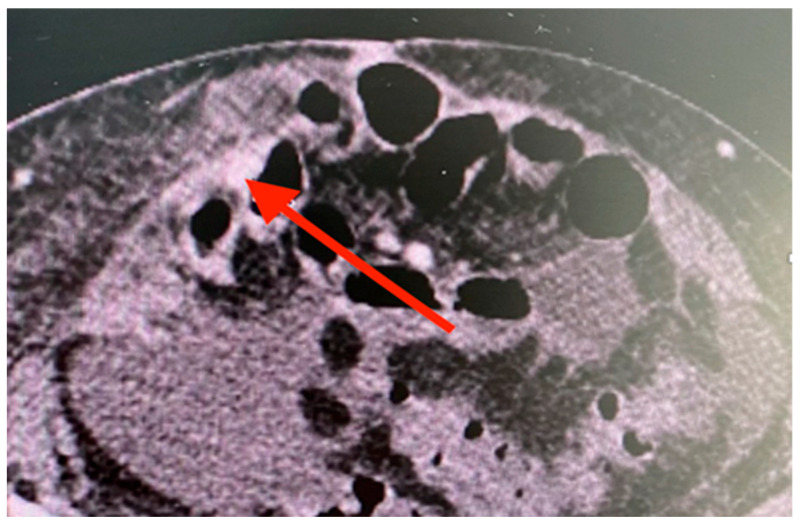
Ileal portocaval shunts. CT angiography image showing portocaval shunt in the ileum (red arrow); no active bleeding site was detected.

Due to the hemodynamically unstable status and signs of active bleeding, we decided to perform CT angiography. No active bleeding site was detected; nevertheless, several portocaval shunts in herniated ileal loops were noted (Figure 1). Afterwards, due to intensive care management and fluid resuscitation, the patient was stable enough to allow us to perform anterograde single balloon enteroscopy. Still, the bleeding spot was not detected.

**Figure 2:**
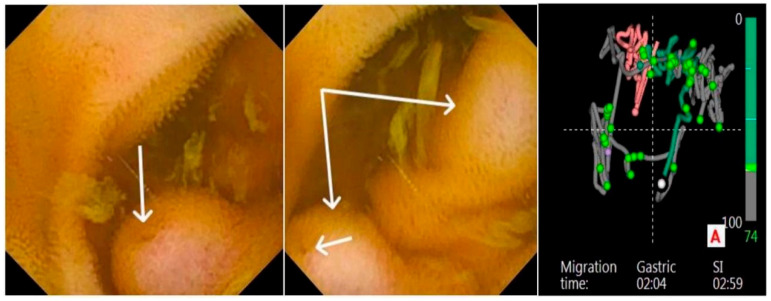
Ileal variceal cords with bleeding stigmata. Capsule endoscopy findings: enteral varices with recent bleeding stigmata—red dots (white arrows, left and middle image); endoscopic capsule software trajectory locator (right image)—the segment highlighted in red indicates the position of the varices—in this case pertaining to the ileum.

We continued to capsule endoscopy and the recordings showed multiple ileal protrusive enteral variceal cords with bleeding stigmata (Figure 2). Moreover, fresh blood was observed at the level of the cecum and proximal colon, which was highly suggestive of a recent bleeding episode which spontaneously ceased.

A transjugular intrahepatic portosystemic shunt (TIPS) was not available in our unit and its expertise, mainly in the acute setting (salvage TIPS), is generally limited in the whole country.

**Figure 3:**
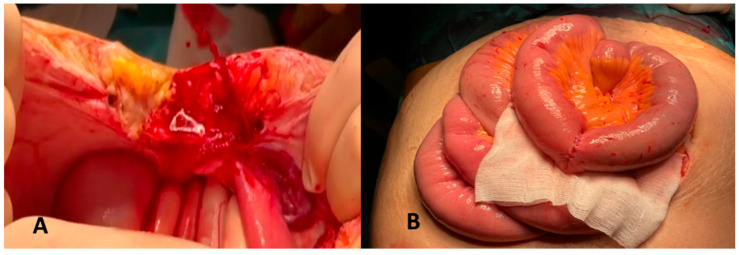
Intraoperative images. Active variceal bleeding during laparotomy (**A**). Successful enterectomy followed by enterorrhaphy (**B**).

Due to the continuously deteriorating clinical status, surgery was considered the last resort and laparotomy identified an active bleeding variceal cord (Figure 3A). Segmental enterectomy and enterorrhaphy was performed with a favorable outcome (Figure 3B). She was discharged 7 days after surgery, in good condition.

## 3. Discussions

Ectopic varices represent a rare and challenging complication, mainly caused by portal hypertension. They are dilated portosystemic collaterals located in unusual sites, different from the gastro-esophageal region, and account for up to 5% of all the variceal bleeding episodes [3]. In a study conducted on 169 patients admitted for bleeding from ectopic varices, those occurring from the small intestine accounted for 17% [4]. In comparison, in another study conducted on 46 patients, 8.7% of varices were located in the small bowel [5].

In spite of the advances in the fields of radiology and endoscopy with both diagnostic and therapeutic yields, it is still difficult to explore the ileo-jejunal varices and to estimate their true burden.

When it comes to the exploration of overt gastrointestinal bleeding, it can be wise to consider second-look endoscopy and/or colonoscopy, since there are randomized controlled trials comparing the outcomes of capsule endoscopy and push enteroscopy, which demonstrated that a significant proportion of patients (up to 36.7%) actually had a bleeding lesion in the stomach, duodenum and colon, regions that are visualized by standard endoscopy [6]. In our case report, both were negative, so we decided to investigate the small bowel, as current guidelines recommend.

Due to the unstable hemodynamic status, we proceeded to CT angiography. It is a fast and reliable examination with an overall 79% sensitivity and 95% specificity in the detection of active GI hemorrhage [7,8]. Intermittent GI hemorrhage is common and performing a CT angiographic examination when the bleeding is active is pivotal in localizing the incriminated lesion [9].

Capsule endoscopy can be the first line procedure as ACG guidelines recommend, with a 92% diagnostic rate in patients with active bleeding if hemodynamically stable [10]. Its main advantage is the capability of exploring the entire small bowel with minimal risks (1% risk of impaction), but there are also drawbacks such as its lack of therapeutic potential and the fact that it may be limited by inadequate bowel preparation, a limited field of view and poor visual clarity [11]. Another limitation of capsule endoscopy is the false-negative results, which are up to 11% in hemorrhagic lesions [12].

Another diagnostic method which poses therapeutic hemostatic maneuvers with a relatively small rate of complication is balloon-assisted enteroscopy, particularly single-balloon enteroscopy [13]. Motorized spiral enteroscopy was used in the past to achieve the complete intubation of the small bowel in the shortest procedure time in clinical trials, with a design offering excellent therapeutic capabilities. However, the device was withdrawn from the global market in 2023, after an unexpected major adverse effect, which led to a post-market risk assessment and the removal of the technique [13,14]. Single-balloon enteroscopy systems are still widely available and used throughout the world.

When it comes to managing bleeding from ectopic varices, there are currently no established guidelines or randomized controlled trials. Therefore, it is recommended to adopt a multidisciplinary approach that includes expertise from gastroenterology, radiology and surgery fields. Management depends on several factors such as location of the hemorrhage, team expertise, available local equipment and the underlying cause [15].

There are various treatment options available for ectopic varices [16], including endoscopic band ligation (EBL) and endoscopic injection sclerotherapy (EIS) using different sclerosing agents [17]. While EBL has been found to be more effective than EIS in terms of lower rebleeding rates, mortality, complications and faster application, it has a higher rate of variceal recurrence compared to sclerotherapy. On the other hand, the latter may cause deep ulceration which can lead to rebleeding stricture formation or perforation [18,19]. To overcome these limitations, a combination of EBL and EIS called endoscopic scleroligation (ESL) has been developed, which has been shown to be superior with decreased recurrence rates [20]. However, there is no established protocol for the use of these modalities in the management of ectopic varices and the choice often depends on the individual situation and expertise [15].

Balloon-occluded retrograde transvenous obliteration (BRTO) is a retrograde shunt occlusion procedure used for controlling gastric variceal bleeding, as opposed to TIPS, which actually creates a shunt [15]. BRTO at the site of an active bleeding will increase the portal hypertension, which translates into a higher risk of variceal bleeding, especially in the esophagus [21]. Consequently, a combination of BRTO and TIPS can be used in order to prevent or to decrease the risk of hemorrhage from other variceal sites [22]. This combined method has a rate of success of 89% without significant side effects regarding hepatic function and without triggering encephalopathy [23]. Recently, case reports of BRTO being successfully used in the treatment of varices in the small bowel have been increasing [24,25]. However, the rebleeding rates are between 5 and 16% in hemorrhagic complications due to ectopic varices [26].

Multiple studies have highlighted the effectiveness of TIPS in managing bleeding ectopic varices in cirrhotic patients caused by intrahepatic portal hypertension. In terms of preventing recurrent esophageal bleeding, TIPS is superior to other endoscopic therapies. A pressure gradient reduction of more than 50% is considered highly protective from rebleeding events and even a reduction of 25–50% can be effective [27]. Following TIPS, the cumulative rate of rebleeding was 23% at 1 year and 31% at 2 years [28].

Percutaneous embolization using a transhepatic approach has been successfully performed in tertiary centers but it carries a very high rebleeding rate (up to 65%) [29]. Percutaneous embolization has been reported for the management of ectopic variceal bleeding but, given the high rebleeding rates, it is advised to be used in combination with other techniques like TIPS [30,31].

In terms of surgery, its role has been diminished in favor of modern management techniques and is nowadays considered a salvage option for patients when endoscopic and radiological procedures failed. Certainly, patients who have ectopic variceal bleedings are in a state of decompensated liver status and this represents a marker of high operative morbidity and mortality. Nevertheless, there are authors who reported successful partial ileal resection of the affected bowel in patients with bleeding varices with no further hemorrhagic events at a minimum of 1 year follow-up [32,33,34,35]. Furthermore, other studies had good results and advocate for a more direct approach with surgical management, even in CHILD-PUGH B or C patients [36,37,38,39,40].

One of the downsides is that resection does not address the causative factor, which is portal hypertension; therefore, a combined technique of enterectomy of the affected bowel and TIPS during the same hospitalization seems superior regarding long-term rebleeding rates. Another argument for this integrated approach would be the fact that 25% of patients with cirrhosis and small intestinal varices had an episode of hemorrhage with frequent need for non-surgical second interventions and a high mortality rate [41].

Currently, the literature does not offer a direction or plan in managing ectopic variceal bleedings and medical decisions should be made based on expert opinion for every case, with an interdisciplinary approach where input is made not only by surgeons, gastroenterologists, radiologist, and intensivists, but also by the primary care physician, who should closely monitor the patient and establish a personalized medical guidance plan.

## 4. Conclusions

Diagnosing and treating ectopic small bowel varices, as a complication derived due to portal hypertension, are demanding tasks, especially in acute settings.

The novelty of this case lies in the rare occurrence of ileal variceal bleeding, a condition seldom encountered in clinical practice, with a lack of established treatment guidelines for such cases. Additionally, the successful management of the patient was facilitated by the efficient and responsible use of advanced diagnostic tools. The endoscopic capsule played a pivotal role in accurately detecting the bleeding when the CT angiography was unable. The exceptional collaboration of specialists from multiple medical fields allowed timely surgical intervention and hemorrhage control, highlighting the importance of interdisciplinary teamwork in addressing such complex cases.

Randomized controlled trials and guidelines are expected to clarify and optimize the proper management.

## Data Availability

The original contributions presented in this study are included in the article. Further inquiries can be directed to the corresponding author.
